# Human norovirus binding to select bacteria representative of the human gut microbiota

**DOI:** 10.1371/journal.pone.0173124

**Published:** 2017-03-03

**Authors:** Erin A. Almand, Matthew D. Moore, Janie Outlaw, Lee-Ann Jaykus

**Affiliations:** 1 Department of Plant and Microbial Biology, North Carolina State University, Raleigh, NC, United States of America; 2 Department of Food, Bioprocessing and Nutrition Sciences, North Carolina State University, Raleigh, NC, United States of America; Universita degli Studi di Parma, ITALY

## Abstract

Recent reports describe the ability of select bacterial strains to bind human norovirus, although the specificity of such interactions is unknown. The purpose of this work was to determine if a select group of bacterial species representative of human gut microbiota bind to human norovirus, and if so, to characterize the intensity and location of that binding. The bacteria screened included naturally occurring strains isolated from human stool (*Klebsiella* spp., *Citrobacter* spp., *Bacillus* spp., *Enterococcus faecium* and *Hafnia alvei*) and select reference strains (*Staphylococcus aureus* and *Enterobacter cloacae*). Binding in PBS was evaluated to three human norovirus strains (GII.4 New Orleans 2009 and Sydney 2012, GI.6) and two surrogate viruses (Tulane virus and Turnip Crinkle Virus (TCV)) using a suspension assay format linked to RT-qPCR for quantification. The impact of different overnight culture media prior to washing on binding efficiency in PBS was also evaluated, and binding was visualized using transmission electron microscopy. All bacteria tested bound the representative human norovirus strains with high efficiency (<1 log_10_ of input virus remained unbound or <10% unbound and >90% binding efficiency) (p>0.05); there was selective binding for Tulane virus and no binding observed for TCV. Binding efficiency was highest when bacteria were cultured in minimal media (<1 log_10_ of input virus remained unbound, so >90% bound), but notably decreased when cultured in enriched media (1–3 log_10_ unbound or 0.01 –<90% bound)) (p<0.05). The norovirus-bacteria binding occurred around the outer cell surfaces and pili structures, without apparent localization. The findings reported here further elucidate and inform the dynamics between human noroviruses and enteric bacteria with implications for norovirus pathogenesis.

## Introduction

Human norovirus is the leading cause of acute viral gastroenteritis worldwide [1], and also the most common cause of foodborne disease, at least within the United States [2]. The public health and economic burden of norovirus infection is substantial in the Western world, and may be crippling in developing countries [3]. Increased awareness of this ubiquitous pathogen has placed considerable interest in developing diagnostics, antivirals and vaccines, as well as finding more effective ways to halt its transmission. Critical to these efforts is elucidating the details of the norovirus infection cycle. Until recently, the lack of a reliable propagation model for human norovirus (reviewed in [4,5]) had been a significant roadblock, and little is still known about the viral replication cycle.

About fifteen years ago, researchers identified human histo-blood group antigens (HBGAs) as a putative human norovirus cellular receptor, [6,7]. HBGAs are complex terminal carbohydrates present on key cellular surfaces (i.e. red blood cells and mucosal epithelium) or secreted into biological fluids (i.e. saliva, intestinal lumen) [8]. Most, but not all, human norovirus strains interact with these sugar moieties in specific binding patterns [9] linked to residues on the A/B or Lewis antigens [10].

Structures similar to human HBGAs are found ubiquitously in other animals, plants, and even in bacteria [8,11]. Recently, Miura et al. [12] demonstrated that *Enterobacter cloacae* spp. cloacae (ATCC 13047) binds to human norovirus GI.1 and GII.6, interactions that appears to be mediated by bacterial HBGA-like moieties. These findings and others [13,14] informed the development of a mammalian cell culture model for human norovirus propagation in which the target cell line (Burkitt’s Lymphoma (BJAB) B cells) could support modest replication of the virus only when in the presence of *E*. *cloacae* or after supplementation with synthetic HBGAs [15]. This system has recently been replicated and used to study a viral polymerase inhibitor [16]. Recently, another report of successful *in vitro* human norovirus replication has been reported in human intestinal enteroids. Interestingly, bacteria was not required for productive infection in this model. However, the dependency upon enteroids derived from secretors (who express HBGAs) for GII.4 Sydney 2012 suggested that HBGAs are involved to some degree in pathogenicity for some strains [17].

Despite this impressive achievement, there is still a poor understanding of the dynamics and importance of bacteria-norovirus interactions. Certainly, HBGAs are not the only compounds implicated in human norovirus binding, and *E*. *cloacae* may not be the only strain to which the virus binds. The studies to date have focused on only a limited number of bacterial strains when, in reality, microbial populations within the human gastrointestinal tract are quite complex. This finding begs the question as to whether human norovirus-bacteria interactions are an isolated occurrence or a more widespread phenomenon. The purpose of this research was to characterize the specificity and intensity of these interactions using a select group of bacterial species representative of human gut microbiota. Both Gram-negative and Gram-positive bacterial strains (reference and fecal isolates) were screened for binding with genotypes GI.6 and GII.4 (New Orleans 2009 and Sydney 2012) human norovirus strains, as well as the cultivable Tulane virus surrogate. Tulane virus is a recently discovered *Calicivirus* that infects the gastrointestinal tracts of rhesus monkeys, and has been shown to selectively bind type A and B HBGAs and sialic acid [18–21]. Additionally Turnip Crinkle Virus, a plant virus whose capsid organization and structure (T = 3, icosahedral symmetry, one major coat protein) closely resembles noroviruses [22] was chosen as a negative control as it does not target HBGAs as receptors. Information was collected on binding efficiency, the impact of growth conditions on bacteria-virus binding, and the location on the bacteria to which the virus bound.

## Results

### Human norovirus binds to bacteria

**Fig 1:**
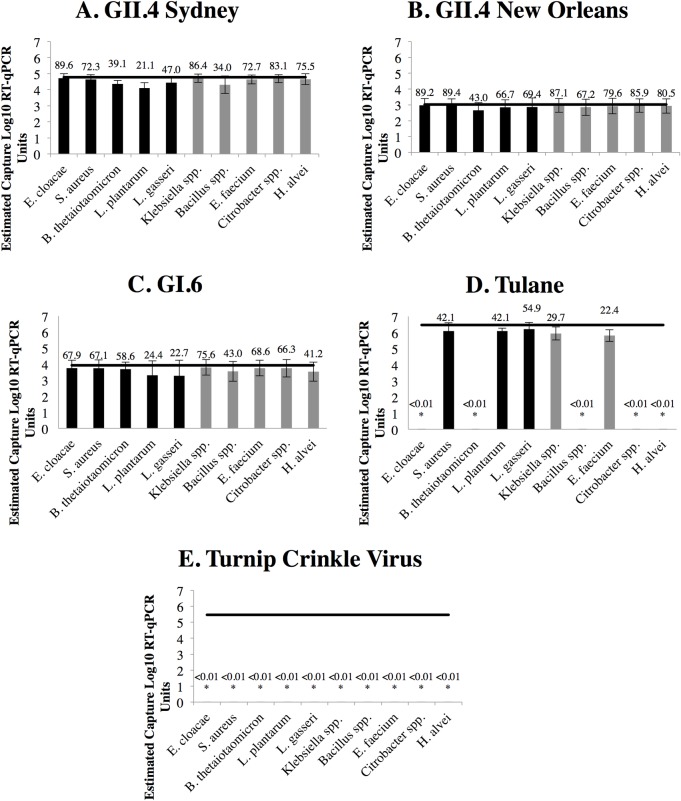
Binding efficiency of human norovirus and representative surrogate viruses to select bacteria. The line indicates the total virus input. Data are expressed as log_10_ mean concentration ± the standard deviation of bacteria bound (in RT-qPCRU) (bars) and percent binding efficiency as determined by loss-to-supernatant ([total input virus-supernatant virus]/total input virus) (numerical). The black bars correspond to ATCC or control strains. The gray bars correspond to bacteria isolated in this study. All bacteria grew anaerobically in TSB with the following exceptions: *B*. *thetaiotaomicron* (chopped meat medium), *L*. *gasseri* (MRS) and *L*. *plantarum* (MRS). Asterisks (*) represent values for which there was a statistically significant difference (p<0.05) between the viral input load and the bacterial capture amount based on log_10_ RT-qPCRU. Data represents averages and standard deviations of the assays performed in triplicate.

Thirteen bacterial species were originally isolated from the GI.6-positive stool sample after growth on Tryptic soy broth (TSB), TSB+5% defibrinated sheep blood (blood), Brucella agar (BA), BA+blood, Brain Heart Infusion (BHI) and/or de Man, Rogosa and Sharpe (MRS) agars under anaerobic conditions. Primary microbial analysis putatively determined identity, and after deletion of duplicates, there were eight unique species. These eight strains were sent for 16S rDNA sequencing. Of the eight isolated, three appeared different physiologically but were duplicates on the genetic level. The five resulting species were evaluated for their ability to grow on different media. Additional reference bacteria were included, i.e., *L*. *gasseri*, *L*. *plantarum* and *B*. *thetaiotaomicron* based on their beneficial role within the human gut and overall prevalence in the gastrointestinal tract [23]. While most of the bacterial isolates were able to grow in all listed media types (Table 1), the lactobacillus and bacteroides species were more fastidious (Table 1), and were only able to be compared across different viruses, not different growth conditions (Fig 1).

**Table 1:** Bacterial strains and growth media used in this study.

Bacterial Strain	Growth Media	Source
*Staphylococcus aureus*	TSB, or as indicated in Fig 2	ATCC® 23235
*Enterobacter cloacae*	TSB, or as indicated in Fig 2	ATCC® 13047
*Bacteroides thetaiotaomicron*	Chopped meat medium	ATCC® 29148
*Lactobacillus plantarum*	MRS	Klaenhammer
*Lactobacillus gasseri*	MRS	Klaenhammer
*Klebsiella* spp.	TSB, or as indicated in Fig 2	This study
*Bacillus* spp.	TSB, or as indicated in Fig 2	This study
*Enterococcus faecium*	TSB, or as indicated in Fig 2	This study
*Citrobacter* spp.	TSB, or as indicated in Fig 2	This study
*Hafnia alvei*	TSB, or as indicated in Fig 2	This study

Suspension assays in PBS were used to evaluate the efficiency of binding of select bacteria to a group of viruses. Collectively, all bacterial strains bound all human norovirus strains with high efficiency, as in all cases <1 log_10_ of input virus was lost to supernatant, or >90% bound (Fig 1). There were no statistically significant differences in binding efficiency when percentages were compared across the three norovirus genotypes, although there was a general trend for better binding for the GII.4 strains vs. the GI.6 strain. There were differences between bacteria binding observed for Tulane virus, for which individual bacterial strains either bound to the virus at a level statistically equivalent to the other norovirus strains, or the bacteria did not bind the virus at all. None of the bacterial strains bound the negative control, Turnip Crinkle Virus (TCV), to any appreciable degree. Taken together, these data suggest that human norovirus-bacteria binding occurs with high efficiency but with relatively low inter-species specificity.

### Human norovirus-bacteria binding efficiency is impacted by culture media

**Fig 2:**
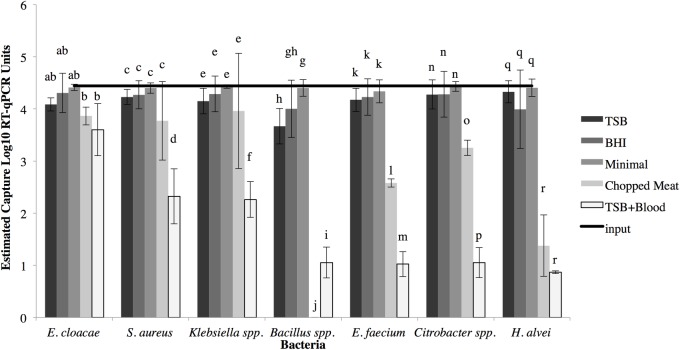
Binding efficiency of the GII.4 Sydney 2012 when bacterial strains were grown in different media. The line indicates the total virus input. Data are expressed as mean log_10_ concentration of bacteria bound ± the standard deviation (in RT-qPCRU) (bars) and percent binding efficiency as determined by loss-to-supernatant ((total input virus-supernatant virus)/total input virus) (numerical). Letters indicate statistically significant differences (p<0.05) between the amount of virus bound for each bacterial strain cultured using different growth media. Different letters within the same bacteria indicate statistical difference. Statistical differences in binding between different bacteria was not tested. Data represent averages and standard deviations of the assays performed in triplicate.

To further elucidate factors impacting the dynamics of bacteria-virus binding, experiments were undertaken in which the bacterial strains were grown in enriched media (chopped meat and TSB+blood), nutrient dense media (TSB and BHI), or minimal media and then equal amounts of bacterial cells assayed for binding in PBS with GII.4 Sydney 2012, the most recent epidemic human norovirus strain. For minimal media or nutrient dense media (TSB or BHI), there were no statistically significant differences between the input virus concentrations and virus concentrations estimated as bound to bacteria for any of the bacterial species, suggesting high capture efficiency (Fig 2). On the other hand, the bacteria-virus binding efficiency notably decreased in enriched media, a phenomenon that was statistically significant (p<0.05) in most cases, and most pronounced for *Bacillus* spp., *E*. *faecium*, *Citrobacter* spp., and *H*. *alvei*. In general, TSB+5% sheep blood had the greatest negative impact on human norovirus binding to bacteria. A minimal media or reduced strength media produced the most consistent, highest degree of binding.

### Human norovirus targets bacterial pili and cell membranes

**Fig 3:**
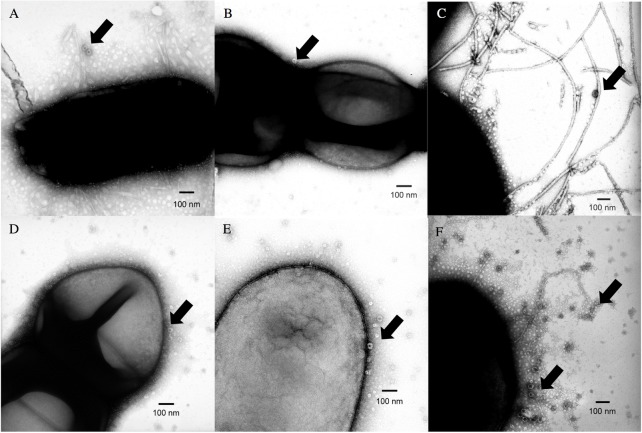
**Transmission electron microscopy (50,000x) photos of select bacteria to which GII.4 Sydney 2012 (A-C) and GII.4 Farmington Hills 2002 (D-F) VLPs are bound.** Bacteria-VLP interactions are shown as follows: (A) *E*. *cloacae*, (B) *S*. *aureus*, (C) *Bacillus* spp., (D) *E*. *faecium*, (E) *Citrobacter* spp., and (F) *H*. *alvei*. Representative VLPs are pointed out with the arrows, although additional VLPs are frequently also present in the image. Images shown are representative of multiple fields of view.

Three Gram-negative and three Gram-positive bacteria (two ATCC strains and four fecal isolates) were viewed using transmission electron microscopy (TEM) after exposure to either GII.4 Sydney 2012 or GII.4 Farmington Hills 2002 virus-like particles (VLPs) that contain just the purified assembled capsid protein (Fig 3A–3C and 3D–3F, respectively). Because of the need for high assay resolution, TEM experiments were done using VLPs in place of fecally-derived virus. Of the bacteria imaged, three (*S*. *aureus* (Fig 3B), *E*. *faecium* (Fig 3D), and *Citrobacter* spp. (Fig 3E)) showed VLPs bound to the outer cell membrane; two (*E*. *cloacae* (Fig 3A) and *Bacillus* spp. (Fig 3C)) had VLPs bound to pili; and one (*H*. *alvei* (Fig 3F)) showed evidence of VLP binding to both structures. For the latter two instances, the binding was scattered around the cell membrane rather than localized to specific structures.

## Discussion

The purpose of this research was to characterize the specificity and intensity of human norovirus interactions with a select group of bacterial species representative of the human gut microbiota. Based on the results of a suspension-RT-qPCR binding assay, we found that naturally occurring human fecally-derived bacterial isolates, as well as repeatedly cultured reference bacteria, were all capable of binding to human norovirus with relatively high efficiency (Figs 1 and 2). These interactions were limited to human norovirus strains as the genotypes tested bound well to all bacteria screened. There were limited interactions between these same bacteria and the Tulane virus surrogate, and no binding was observed between any of the bacteria and the unrelated TCV. In short, virus-bacteria binding was specific at the norovirus genus level, but relatively promiscuous when considering the bacterial species. In addition, the presence of bacteria in the human stool matrix did not appear have a considerable effect on the virus-bacterial binding interaction (data not shown), which would not be surprising because the viral inoculum was diluted 1/500 (thus 0.2% stool) prior to use in the binding assay.

The method used in these studies provided results supporting norovirus-bacteria binding, but did not provide information on how or why that binding occurred. Perhaps the most logical explanation for this binding is the expression of HBGA-like moieties by bacterial cells [11]. There is recent evidence of a naturally-occurring *E*. *cloacae* strain that readily bound to human norovirus, and HBGA (specifically, A type)-like moieties in the bacterial EPS were associated with that binding [22]. Li et al. [23] screened multiple bacterial strains for norovirus VLP binding, finding that virus binding of the bacteria correlated well with their HBGA expression profiles. In an effort to identify HBGA-type molecules in the bacteria we studied, the same assays used by these previous investigators were applied here, in addition to a previously-established ELISA assay [24]. We found all three of the assays difficult to reproduce and/or interpret (data not shown). Whether this challenge was a function of the quality and/or source of the reagents, minor differences in assay design, or simply a lack of HBGA-like activity of the bacteria is unknown. We therefore have no concrete evidence on the definitive mechanism(s) responsible for the observed virus-bacteria binding.

There is, however, some evidence supporting a potential role for bacterial HBGA-like molecules in human norovirus binding. For example, Tulane virus bound to some but not all bacterial strains tested. Tulane is genetically related to human norovirus and binds HBGAs in a highly selective manner (i.e., B and H antigens as well as sialoglycoconjugates), overlapping receptors with some human norovirus strains [25]. The failure of TCV—a genetically unrelated plant virus of similar size, shape, and charge to human norovirus [26]—to bind to any of the bacteria suggests that human norovirus-bacteria binding is more likely to be associated with a receptor-ligand (HBGA) interaction rather than charge-based or indirect interactions. Differences in bacteria-virus binding efficiency as a consequence of media formulation is also supportive of this, as differential expression of many bacterial genes occurs as a consequence of growth conditions [27]. It is possible that poor virus-bacteria binding efficiency in the presence of high nutrient composition media could be a consequence of binding site occlusion, however bacterial cells were washed and diluted prior to binding assays. Taken together, we believe the data presented here provides indirect evidence that a common ligand, like HBGA-like molecules, mediates virus-bacteria interactions.

In addition to HBGA-like molecules, bacteria possess sialylated gangliosides commonly implicated in virus binding [25,28]. These sialylated lipopolysaccharides are a major component of the outer surface of Gram-negative bacteria, on molecules which are also associated with histo-blood group activity (i.e. galactose) [29]. In the same binding pocket as HBGAs and sialic acid, human norovirus has been shown to bind citrate. This molecule is comprised of a pyranoside ring, which closely resembles, and in this case may mimic, a terminal fucose characteristic of HBGAs [19]. As citric acid is an intermediate in the tricarboxylic acid cycle, it is possible a surplus of this molecule is also aiding in norovirus binding to different bacteria [29]. While the exact molecule responsible for norovirus binding to bacteria has yet to be conclusively identified, the evidence we and others present suggests specific binding motifs rather than nonspecific, indiscriminate binding.

Unlike the findings of Miura et al. [12], who reported norovirus binding occurred to the bacterial exopolysaccharide matrix, we were unable to observe consistent VLP binding in the EPS. It should be noted that the negative staining used to prepare samples for electron microscopy made it difficult to visualize the external structures of the bacteria without compromising the ability to see the VLPs. Thus, it is possible that additional localized interactions are occurring between the virus and the bacteria in the EPS that were not visible due to methodological limitations. However, we did visualize binding to different bacterial structures, specifically cell membranes and pili, consistently. These structures on the outside of the cell may help mediate adhesion, and/or may also camouflage the bacteria from the host to prevent interactions that could be detrimental to the bacteria or the virus. From the virus’ perspective, adhering to the pili might place it closer to host cell binding regions, facilitating infection.

The impact of growth conditions on the norovirus-bacteria binding is intriguing. Mechanistically, one could make the argument that the small intestine is a “nutrient rich” environment, in which case lower degrees of bacteria-virus binding might be expected. On the other hand, the nutrient dense times in this organ are short (approximately 1–2 h [30]) and are separated by long periods devoid of nutrients. Since the bacteria would do the majority of their growth in periods with low nutrient density, it is possible that expression of binding ligands might be up-regulated during fasting. It is possible that the bacteria were competing for binding with residual HBGA-like substances in the richer blood and chopped meat media, however the cells were washed once with PBS and no difference in binding ability was seen with additional washes. Further, the washed bacteria were diluted 1/5 in a different tube for performing the suspension assay. However, there are many other factors involved (e.g., pH, temperature, bacterial interactions) that could influence bacteria-virus binding, and given the effect of growth medium we report, further elucidation of their effect on viral binding would be a logical future direction for investigation [27].

Improved understanding of norovirus-bacterial interactions has widespread implications ranging from understanding virus infectivity [13,14]; aiding in *in vitro* cultivation of human norovirus [15]; developing concentration and purification methods for detection [12]; and design of novel removal/inactivation methods [31,32]. Regardless of potential downstream applications, the phenomenon of bacteria and virus interaction resulting in advantages to one or both of the infectious entities is being increasingly recognized. The impact of this shifting paradigm will need to be considered in many different aspects of human norovirus study, providing fruitful avenues for further research and applications. The data we report here builds on and advances the increasingly important field of human norovirus-bacteria interactions.

## Materials and methods

### Virus strains and virus-like particles (VLPs)

Human fecal specimens derived from outbreaks and confirmed positive (by sequencing) for GI.6, GII.4 New Orleans 2009, and GII.4 Sydney 2012 norovirus were obtained courtesy of Dr. Shermalyn Greene, North Carolina State Laboratory of Public Health, Raleigh, NC. Stool samples were diluted to 20% (v/v) in 1X PBS, pH 7.2 (Life Technologies, Carlsbad, CA), aliquoted, and stored at -80°C until use. Tulane virus, (obtained courtesy of Dr. Jason Jiang, Cincinnati Children’s Hospital Medical Center, Cincinnati, OH) was cultivated in the LLC-MK2 cell line (ATCC CCL7, American Type Culture Collection, Manassas VA) as previously reported [19], and harvested by three cycles of freeze-thaw at -80°C followed by centrifugation. Aliquots of the virus-rich supernatant were stored at -80°C. A purified stock of Turnip Crinkle Virus (TCV) was obtained from the laboratory of Dr. Steven Lommel (North Carolina State University, Raleigh, NC) and diluted in PBS. Virus-like particles (VLPs) for strains GII.4 Sydney 2012 (1.0mg/ml) and GII.4 Farmington Hills 2002 (1.3mg/ml) were provided courtesy of Dr. Robert Atmar (Baylor College of Medicine, Houston, TX).

### Bacterial isolates

Bacterial strains used in this study are shown in Table 1. Stock cultures of *Lactobacillus plantarum* and *Lactobacillus gasseri* were obtained courtesy of Dr. Todd Klaenhammer (North Carolina State University, Raleigh, NC). Reference strains, provided by the American Type Culture Collection (ATCC; Manassass, VA) included *Staphylococcus aureus* (ATCC 25235), *Enterobacter cloacae* (ATCC 13047) and *Bacteroides thetaiotaomicron* (ATCC 29148). To obtain natural bacterial isolates, human stool from one patient positive for GI.6 norovirus was diluted and plated in 100μl aliquots onto tryptic soy agar (TSA; Thermo Fisher Scientific, Waltham, MA); TSA supplemented with 5% defibrinated sheep blood (Lampire Biological Products, Pipersville, PA); Brucella agar (BA; Thermo Fisher Scientific); and BA supplemented with 5% defibrinated sheep blood. For the lactobacillus species, de Man, Rogosa and Sharpe agar or broth (MRS; Thermo Fisher Scientific) was used for cultivation. These plates were incubated anaerobically (5% carbon dioxide, 10% hydrogen, 85% nitrogen) in an anaerobic glove box with CAM-12 oxygen monitoring system and heating unit (Coy Laboratories, Grass Lake, MI) for 24 h at 37°C. Unique colonies from each agar plate (i.e. size, color, shape, hemolysis) were isolated, purified, and grown anaerobically at 37°C overnight on both the agar and in the broth formulations from which they were initially isolated. Stocks were made in 30% glycerol and stored at -80°C. Each of the isolated fecally derived cultures underwent further characterization using Gram stain, motility tests and the Oxoid Microbact GNB 24E reagent system (Thermo Fisher Scientific). To identify the bacteria, whole agar plates were sent and a single colony of each bacterial isolate was sequenced via 16S rDNA by GENEWIZ (South Plainfield, NJ) (S1 Fig). Bacterial IDs were confirmed using NCBI Blast (S1 Table).

### Bacteria-virus binding assays

The ability of bacteria to bind norovirus was modified from the plate-based assay of Miura et al [12] to a suspension test design. Since preliminary studies showed that bacteria-virus binding was highly dependent upon initial bacterial growth conditions (data not shown), several different media formulations were used for bacterial propagation. Specifically, for minimal media, two formulations were used. The Gram-negative medium consisted of M9 minimal salts (supplemented with 20% glucose, 0.2% MgSO_4_ and 0.01% CaCl_2_; Sigma-Aldrich, St. Louis, MO), while the Gram-positive bacteria were grown in one-half strength Tryptic Soy Broth (TSB). On the other end of the nutrient spectrum both Gram-negative and Gram-positive bacteria (*E*. *cloacae*, *S*. *aureus* and the fecal isolates) were grown in two rich media types typically used to cultivate fastidious microbes, i.e., chopped meat broth (Anaerobe Systems, Morgan Hill, CA) and TSB supplemented with defibrinated sheep blood (Lampire Biological Laboratories, Pipersville, PA). Prior to experimentation, starting concentrations (input) of bacteria were enumerated for each bacterial strain and media combination via growth curves to ensure that comparable amounts of bacteria were used in each binding assay.

Prior to binding assays, bacterial cultures were grown anaerobically for 24 h at 37°C in 10 ml of select medium. The cells were then pelleted, washed once, and resuspended in 10 ml PBS, pH 7.2. These stocks were diluted to concentrations of approximately 1x10^7^cfu/ml. Human norovirus suspensions were diluted 100-fold in PBS to concentrations around 1x10^5^ RT-qPCR amplifiable units (RT-qPCRU)/ml (GII.4 Sydney 2012), 1x10^3^ RT-qPCRU /ml (GII.4 New Orleans 2009) and 1x10^4^ RT-qPCRU /ml (GI.6). Tulane virus and TCV were likewise diluted in PBS to reach a final concentration of 1x10^7^ RT-qPCRU /ml and 1x10^6^ RT-qPCRU/ml, respectively.

For each suspension assay, 100μl of resuspended bacteria was diluted in 300μl PBS, and 100μl of diluted virus suspension was added. The mixture was incubated for 2 h at 37°C with rotation. The mixture was pelleted at 10,000 *x g* for 5 min at room temperature and the supernatant was removed for enumeration of remaining (unbound) viruses using RT-qPCR. The pellet was also retained for comparison. Positive controls consisted of input virus suspension without exposure to bacteria; negatives controls consisted of PBS alone.

### Nucleic acid extraction and detection of viral RNA

The bacterial supernatants and in some instances, pellets, were processed for RNA extraction using the NucliSENS^®^ easyMAG automated system (bioMérieux SA, Marcy l’Etoile, France) as per manufacturer instructions, with a final resuspension volume of 50μl.

Detection of viral RNA was carried out by Reverse Transcriptase quantitative PCR (RT-qPCR) using primers and probes outlined in Table 2. The 25μl reaction consisted of 12.5μl 2X reaction buffer (SuperScript® III One-Step qRT-PCR Kit, Invitrogen, Grand Island, NY), 0.5μl RT/Platinum® Taq Mix, 400nM forward and reverse primers (Table 2; Integrated DNA Technologies, Coralville, IA), 200nM fluorescently labeled TaqMan probe (Table 2; Integrated DNA Technologies) and 2.5μl of RNA. A CFX96 Touch™ Real Time PCR Detection System (Bio-Rad, Hercules, CA) thermocycler was used with the following amplification conditions: (1) reverse transcription for 30 min at 50°C; (2) denaturation for 15 min at 95°C; and (3) 45 cycles of 15 s at 95°C then 30 s at 60°C. All primers and probes utilized the same amplification protocol.

**Table 2:** Primers and probes used for RT-qPCR detection of viruses in this study.

Pathogen	Primer/Probe Name	Sequence (5’-3’)	Reference
**GI.1 Norwalk**	COG1F	CGYTGGATGCGNTTYCATGA	[33]
	COG1R	CTTAGACGCCATCATCATTYAC	
	RING1a	6-FAM-AGATYGCGATCYCCTGTCCA-BHQ1	
	RING1b	6-FAM-AGATCGCGGTCTCCTGTCCA-BHQ1	
**GII.4 New Orleans 2009/Sydney 2012**	JJV2F	caagagtcaatgtttaggtggatgag	[33,34]
	COG2R	TCGACGCCATCTTCATTCACA	
	RING2P	6-FAM-TGGGAGGGCGATCGCAATCT-BHQ1	
**Tulane Virus**	TV2F	GAGATTGGTGTCAAAACACTCTTTG	[35]
	TV2R	ATCCAGTGGCACACACAATTT	
	TVP	6-FAM-AGTTGATTGACCTGCTGTGTCA-BHQ1	
**Turnip Crinkle Virus**	TCV900F	GTTCGACGCATCTTCCATATCT	[26]
	TCV900R	CTCTTTCCATCAACCCTCTTCTC	
	TCV900Probe	6-FAM-TGGGCAATGGTTTAGACTTTGGAGTCC-BHQ1	

RT-qPCR standard curves for quantification were constructed (in triplicate) as previously described [26] using ten-fold serial dilutions of viral RNA in DEPC-treated water. The CT value corresponding to each serial dilution was plotted and the data analyzed using linear regression to determine a slope. The lower limit of detection for a given standard curve was considered the lowest dilution at which all three replicates were positive for viral RNA; this was designated as 1 RT-qPCRU. The virus input concentration (in RT-qPCRU) before exposure to the bacteria, and that in the supernatant after exposure to the bacteria, were estimated by comparison to the standard curve. Because the amplifications associated with the viruses bound to the pellet were so variable (potentially due to PCR interference associated with excessive amounts of background DNA, data not shown), virus binding efficiency was computed based on loss to supernatant, i.e., ((Total virus input-virus lost in supernatant)/total virus input), expressed as a percentage [36].

### Transmission electron microscopy (TEM)

The bacteria-virus binding experiments were done as described above except for the use of 10μg of VLP (GII.4 Sydney 2012 or GII.4 Farmington Hills 2002). After the bacteria-virus mixture was pelleted, the precipitate was washed and resuspended in 500μl 20mM HEPES (Life Technologies, Carlsbad, CA). Fifteen μl of each sample was applied to 400-mesh carbon-nickel coated grids (Ladd Research, Williston, VT) for 10 min and the excess sample removed using Whatman filter paper (Grade 2, 8μm pore size, Sigma-Aldrich, St. Louis, MO). Negative staining was done with 10μl 2% aqueous uranyl acetate for 60 s. Negative controls consisted of bacteria without exposure to VLPs. Images were visualized using the JEOL 1210 transmission electron microscope (JEOL-USA, Inc., Peabody, MA) at 80 kV at the Center for Electron Microscopy (North Carolina State University, Raleigh, NC).

### Statistical analysis

Three independent experiments were performed for each binding assay. Binding efficiency data were expressed as described above, and also as ± standard deviations across the experiments, depicted by error bars. Statistical comparisons between the log_10_ concentrations of unbound virus based on different bacterial strains (Fig 1) or media type (Fig 2) were done in JMP 11 (SAS Institute, Cary, NC) using the Tukey-Kramer honest significant difference test and considered significant if *p* <0.05.

## Supporting information

S1 Fig Raw 16S sequence information for bacteria isolated from human stool. Data is provided for both forward and reverse reads and isolate names refer to isolates described in S1 Table. Raw forward and reverse reads of the isolates relevant to this paper are provided in FASTA format below.
(PDF)

S1 Table Top matches for 16S rRNA sequences of bacteria isolated from human stool samples. Different samples of human stool were streak plated and grown in both aerobic and anaerobic conditions. Specific colonies were isolated, further cultured and their 16S rRNA region sequenced. Below is a summary table of the top sequence isolates selected for further analysis.
(PDF)
