# Supplementary material for: Human norovirus binding to select bacteria representative of the human gut microbiota
Source: PLoS One. 2017 Mar 3;12(3):e0173124. doi: 10.1371/journal.pone.0173124 (PMC5336261; doi:10.1371/journal.pone.0173124)
Supplement: S1 Fig — Data is provided for both forward and reverse reads and isolate names refer to isolates described in S1 Table. Raw forward and reverse reads of the isolates relevant to this paper are provided in FASTA format below. (PDF) [file pone.0173124.s001.pdf]

S1 Fig. Raw 16S sequence information for bacteria isolated from human stool. Data is provided for both forward and reverse reads and isolate names refer to isolates described in Table S1. Raw forward and reverse reads of the isolates relevant to this paper are provided in FASTA format below.

>BA2_ForwardRead
NNNNNNNNNNNNNNNNGNNNNNNNNGTACAGGTAGCCGNNNNTCGTAACAAGGTAGCCGTAA
GTCGTAACAAGGTAGCCGTAAGTCGTAACAAGGTAGCCGTAAGTCGTAACAAGGTAGCCGT
AAGTCGTAACAAGGTAGCCGTAANTCGTAACAAGGTAGCCGTAAGTCCTAACAAGGTAGCC
GTAAGTCGCGCTTTTGNGTCGTGANGGATGGACCGGCGGCGCATTACCTAGTTGGGGAGGTA
ACGGCTCACCAAGGGAACGATGCATAGCCGACCTGAGAGGGTGATCGGCCACATTGGGACT
GAGACACGGCCCAAACTCCTACGGGAGGCAGCAGTAGGGAATCTTCGGCAATGGACGAAAG
TCTGACCGAGCAACGCCGCGTGAGTGAAGAAGGTTTTCGGATCGTAAAACTCTGTTGTTAGA
GAAGAACAAGGATGAGAGTAAAATGTTCATCCCTTGACGGTATCTAACCAGAAAGCCACGG
CTAACTACGTGCCAGCAGCCGCGGTAATACGTAGGTGGCAAGCGTTGTCCGGATTTATTGGG
CGTAAAGCGAGCGCAGGCGGTTTCTTAAGTCTGATGTGAAAGCCCCCGGCTCAACCGGGGA
GGGTCATTGGAAACTGGGAAACTTGAGTGCAGAAGAGGAGAGTGGAATTCCATGTGTAGCG
GTGAAATGCGTAGATATATGGAGGAACACCAGTGGCGAAGGCGGCTCTCTGGTCTGTAACT
GACGCTGAGGCTCGAAAGCGTGGGGAGCAAACAGGATTAGATACCCTGGTAGTCCACGCCG
TAAACGATGAGTGCTAAGTGTTGGAGGGTTTCCGCCCTTCAGTGCTGCAGCTAACGCATTAA
GCACTCCGCCTGGGGAGTACGANCGCAAGGTTGAAACTCAAAGGAATTGACGGGGGCCCGC
ACAAGCGGTGGAGCATGNNGGTTTAATTCGAAGCAACGCGAANAACCTTACCAGGTCTTGA
CNTCCTTTGACCACNNCTAGAGATAGAGCTTCCCCTTCGGGGGCAAAGTGACNGGNGNTGC
ATGGNTNNTCGTCAGCNNNNNGTCGNNGAGANGNNNGGNNAAGTCCCGCAACNNNNCGNN
NCCCTTNTNNNNNNNCNTCATTTANNNNNNCNNNNANNNNANNACNGCNNNGANNNANNN
GNNNNNNNGGNANNANGTNAANCATCNNNCNNNNNNNNNNNNGNNNNNNNNNNNCTNNNNN
NGNNNNNNNNNNNNNNANNNNNNNNNANNNNCNNNNGNNGNNTNNNANCCN

>BA2_ReverseRead
NNNNNNNNNANNNGNNNNGNNNNNNNNNGCNNCNNNNNNNNNACTANNNGCTACCTTGTTAC
GACNNAACGGCTACCTTGTTACGACTTACGGCTACCTTGTTACAACTTACGGCTACCTTGTTA
CGACTTACGGTGACCCGGGATTACTATAGATTCCGGCTGCATGTAGGCAAGTTGCAGCCGAC
AATCCGAACTGAGAGAAGCTTTAAGAGATTAGCTTAGCCTCGCGACTTCGCGACTCGTTGTA
CTTCCCATTGTAGCACGTGTGTAGCCCAGGTCATAAGGGGCATGATGATTTGACGTCATCCC
CACCTTCCTCCGGTTTGTCACCGGCAGTCTCGCTAGAGTGCCCAACTAAATGATGGCAACTA
ACAATAAGGGTTGCGCTCGTTGCGGGACTTAACCCAACATCTCACGACACGAGCTGACGAC
AACCATGCACCACCTGTCACTTTGCCCCCGAAGGGGAAGCTCTATCTCTAGAGTGGTCAAAG
GATGTCAAGACCTGGTAAGGTTCTTCGCGTTGCTTCGAATTAAACCACATGCTCCACCGCTT
GTGCGGGCCCCCGTCAATTCCTTTGAGTTTCAACCTTGCGGTCGTACTCCCCAGGCGGAGTG
CTTAATGCGTTAGCTGCAGCACTGAAGGGCGGAAACCCTCCAACACTTAGCACTCATCGTTT
ACGGCGTGGACTACCAGGGTATCTAATCCTGTTTGCTCCCCACGCTTTCGAGCCTCAGCGTC
AGTTACAGACCAGAGAGCCGCCTTCGCCACTGGTGTTCCTCCATATATCTACGCATTTCACC
GCTACACATGGAATTCCACTCTCCTCTTCTGCACTCNAGTTTCCCAGTTTCCAATGACCCTCC
CCGGTTGAGCCGGGGGCTTTCACATCAGACTTAANAAACCGCCTGCGCTCGCTTTACGCCCA
ATAAATCCGGACAACGCTTGCCACCTACGTANTACCGCGGCTGCTGGNACGTAGTTAGCCGT
GGCTTTCTGGTTAGATACCGTCNAGGGNTGAACATTTTACTCNCATCNNNNNNNCNCTAACA
ANNNNANTTTNACGATCCGAAANNNTNNNNNNNNNNNNNNNGNNNNGCNNNNNNNANTTNN
NNCNNNCNAANANNCNNNNGCTNCNNCNNNANNNTNNGNNNNNTCNCNNNNNAANNNNG
NCNAANCNC

>BAB1_ForwardRead

NNNNNNNNNNNNNNGNANANNNNNNNNGGTANCCGTAGNCNTAACANGGTGNTCGTANNN
CTTAACGGGTGACCGTAGATTAACNCGTGGGTAACCTGCCCCTAAGACTGGGATAACTCCGG
GAAACCGGGGCTAATACCGGATAACATTTTGAACTGCATGGTTCGAAATTGAAAGGCGGCTT
CGGCTGTCACTTATGGATGGACCCGCGTCGCATTAGCTAGTTGGTGAGGTAACGGCTCACCA
AGGCAACGATGCGTAGCCGACCTGAGAGGGTGATCGGCCACACTGGGACTGAGACACGGCC
CAGACTCCTACGGGAGGCAGCAGTAGGGAATCTTCCGCAATGGACGAAAGTCTGACGGAGC
AACGCCGCGTGAGTGATGAAGGCTTTCGGGTCGTAAAACTCTGTTGTTAGGGAAGAACAAG
TGCTAGTTGAATAAGCTGGCACCTTGACGGTACCTAACCAGAAAGCCACGGCTAACTACGTG
CCAGCAGCCGCGGTAATACGTAGGTGGCAAGCGTTATCCGGAATTATTGGGCGTAAAGCGC
GCGCAGGTGGTTTCTTAAGTCTGATGTGAAAGCCCACGGCTCAACCGTGGAGGGTCATTGGA
AACTGGGAGACTTGAGTGCAGAAGAGGAAAGTGGAATTCCATGTGTAGCGGTGAAATGCGT
AGAGATATGGAGGAACACCAGTGGCGAAGGCGACTTTCTGGTCTGTAACTGACACTGAGGC
GCGAAAGCGTGGGGAGCAAACAGGATTAGATACCCTGGTAGTCCACGCCGTAAACGATGAG
TGCTAAGTGTTAGAGGGTTTCCGCCCTTTAGTGCTGAAGTTAACGCATTAAGCACTCCGCCT
GGGGAGTACGGCCGCAGGCTGANCTCANNGAATTGACGGGGGCCCGCANAGCGGTGGNA
NCATGNGGTTTAATTCGAAGCAACGCNNNAANCCTTACCAGGTCNNNACNTCCNCTGAAAC
CCTAGANATNGGGCNNNNNCTNNNGGANCANANNGNNNGGNNNNGCANNNNNTCGTCANC
NNNNNNCGNGAGATGTNNGNNNNTCCCGCANNNNNNNCNANCNNNNNNNNNTNNTTNCNNCN
NNNNNNNNCNNNNAGNNNCNGNCGNNNNNNNNNNNNGANGAAGGNNNGGGNNNNANNNNNN
NNNN

>BAB1_ReverseRead
NNNNNNNNNNNNNNNNGNAANANNNNNNGCACNNGTNCNACTAAGNGCTACCTTGTTACGA
CTTAGTGATACCTTGNTACGACGTACCGGGAGGTGGGTACAACGNACGGGTACGTATTCACC
GCGGANTGCTGATCCGCGATTACTAACGATTCCAGCTTCATGTAGGCGAGTTGCAGCCTACA
ATCCGAACTGAGAACGGTTTTATGAGATTAGCTCCACCTCGCGGTCTTGCAGCTCTTTGTACC
GTCCATTGTAGCACGTGTGTAGCCCAGGTCATAAGGGGCATGATGATTTGACGTCATCCCCA
CCTTCCTCCGGTTTGTCACCGGCAGTCACCTTAGAGTGCCCAACTTAATGATGGCAACTAAG
ATCAAGGGTTGCGCTCGTTGCGGGACTTAACCCAACATCTCACGACACGAGCTGACGACAAC
CATGCACCACCTGTCACTCTGCTCCCGAAGGAGAAGCCCTATCTCTAGGGTTTTCAGAGGAT
GTCAAGACCTGGTAAGGTTCTTCGCGTTGCTTCGAATTAAACCACATGCTCCACCGCTTGTGC
GGGCCCCCGTCAATTCCTTTGAGTTTCAGCCTTGCGGCCGTACTCCCCAGGCGGAGTGCTTA
ATGCGTTAACTTCAGCACTAAAGGGCGGAACCCTCTAACACTTAGCACTCATCGTTTACGGC
GTGGACTACCAGGGTATCTAATCCTGTTTGCTCCCCACGCTTTCGCGCCTCAGTGTCAGTTAC
AGACCAGAAAGTCGCCTTCGCCACTGGTGTTCCTCCATATCTCTACGCATTTCACCGCTACAC
ATGGAATTCCACTTTCCTCTTCTGCACTCNAGTCTCCCAGTTTCCNATGANCCTCCACGGTTG
AGCCGTGNCTTTCACATCANACTTAAGAAACCACCTGCGCGCGCTTTACNCCCAATAATTCC
GNATAACGCTTGCCANCNACGTATTACCNNGGCTGCTGGCACGTANTTAGCCGNNNNNTTCT
GGNTAGGTACCGTNNNGNNNCCAGCTTANTCAANTANCANTNNTTCNTNCNNNACNNNNNA
ANTTTTACNANCCGAANNNNCNTCNNTCANGCNGNNNNCTCNTCNNANTTTCNTCNTGNNN
NANTNCCCTNNNGNNNCNTNNNNNAGNNNNNNNNNNN

>BAB2_ForwardRead
NNNNNNNNNNNNNNNNNNCNCNTGNCNNNNNAACGGTAGCACAGAGGAGCGTTGCTCCTA
TGGGTGACGAGTGGCGGACGGGTGAGTAATGTCTGGGAAACTGCCCGATGGAGGGGGATAA
CTACTGGAAACGGTAGCTAATACCGCATAACGTCGCAAGACCAAAGAGGGGGACCTTCGGG
CCTCTTGCCATCGGATGTGCCCAGATGGGATTAGCTAGTAGGTGGGGTAACGGCTCACCTAG
GCGACGATCCCTAGCTGGTCTGAGAGGATGACCAGCCACACTGGAACTGAGACACGGTCCA
GACTCCTACGGGAGGCAGCAGTGGGGAATATTGCACAATGGGCGCAAGCCTGATGCAGCCA

TGCCGCGTGTATGAAGAAGGCCTTCGGGTTGTAAAGTACTTTCAGCGAGGAGGAAGGTGTTG
TGGTTAATAACCGCAGCAATTGACGTTACTCGCAGAAGAAGCACCGGCTAACTCCGTGCCAG
CAGCCGCGGTAATACGGAGGGTGCAAGCGTTAATCGGAATTACTGGGCGTAAAGCGCACGC
AGGCGGTCTGTCAAGTCGGATGTGAAATCCCCGGGCTCAACCTGGGAACTGCATCCGAAACT
GGCAGGCTAGAGTCTTGTAGAGGGGGGTAGAATTCCAGGTGTAGCGGTGAAATGCGTAGAG
ATCTGGAGGAATACCGGTGGCGAAGGCGGCCCCCTGGACAAAGACTGACGCTCAGGTGCGA
AAGCGTGGGGAGCAAACAGGATTAGATACCCTGGTAGTCCACGCCGTAAACGATGTCGACT
TGGAGGTTGTGCCCTTGAGGCGTGGCTTCCGGAGCTAACGCGTTAAGTCGACCGCCTGGGGA
GTANGGCCGCAAGGTTAAAACTCAAATGAATTGANGGGGCCCGCACAAGCGGTGGANCATG
NGGTTTAATTCGATGCAACGCGAAGAACCTTACCTACTCTTGACNTCCAGANANTTTGCAGA
GATGCGAACGTGCNNNCGGGANTCTGANAANNGGTGCTGCATGGNTGTCGTCAGCTCNTNN
NNGAAATGNTNGGNNNANTCCNNCANNANCGCNNCCNNNCNNNNNCNNNNNNTCGNCNGG
ANTNNANGGNNACTNCNNNNNNANNNNNNNNNGGGNNNANNTNANNNCNNCNNNNNNNN
NNNNGNTANCNCNNNNNANANNGNNNNNNNNNNNNNNNNGNNNNNN

>BAB2_ReverseRead
NNNNNNNNNNNNNNNNNANANNNNNNTNNNCGNNNTCNCGAANAGTTAAGCTACCTACTTC
TTTTAGCAACCCACTCCCATGGTGTGACGGGCGGTGTGTACAAGGCCCGGGAACGTATTCAC
CGTGGCATTCTGATCCACGATTACTAGCGATTCCGACTTCATGGAGTCGAGTTGCAGACTCC
AATCCGGACTACGACATACTTTATGAGGTCCGCTTGCTCTCGCGAGGTCGCTTCTCTTTGTAT
ATGCCATTGTAGCACGTGTGTAGCCCTACTCGTAAGGGCCATGATGACTTGACGTCATCCCC
ACCTTCCTCCAGTTTATCACTGGCAGTCTCCTTTGAGTTCCCGACCGAACCGCTGGCAACAAA
GGATAAGGGTTGCGCTCGTTGCGGGACTTAACCCAACATTTCACAACACGAGCTGACGACA
GCCATGCAGCACCTGTCTCAGAGTTCCCGAAGGCACGTTCGCATCTCTGCAAACTTCTCTGG
ATGTCAAGAGTAGGTAAGGTTCTTCGCGTTGCATCGAATTAAACCACATGCTCCACCGCTTG
TGCGGGCCCCCGTCAATTCATTTGAGTTTTAACCTTGCGGCCGTACTCCCCAGGCGGTCGACT
TAACGCGTTAGCTCCGGAAGCCACGCCTCAAGGGCACAACCTCCAAGTCGACATCGTTTACG
GCGTGGACTACCAGGGTATCTAATCCTGTTTGCTCCCCACGCTTTCGCACCTGAGCGTCAGTC
TTTGTCCAGGGGGCCGCCTTCGCCACCGGTATTCCTCCAGATCTCTACGCATTTCACCGCTAC
ACCTGGAATTCTACCCCCCTCTACAAGACTCTAGCCTGCCAGTTTCGGATGCAGTTCCCAGGT
TGAGCCCGGGGATTTCACATCCGACTTGACAGACCGCCTGCGTGCGCTTTACGCCCAGTAAN
TCCNANTAACGCTTGCANCCTCCGTANTACCGCGGCTGCTGGNACGGAGTTAGCCGGNNNTN
NTTCTGCNANTACGTCANNNNNGNNNNATTNNNNNNNNNNNNNNNNNNCTGAANTACTTANAC
CGANNNNTCATACNNNNGNANGNTGCATCAGGCTGNNNNNTGNGCANNNTNCNNNNGNNN
CNNNCNNNANNNNNNNNGNNCNNNNNNNNNCANNNNNNGNNNNNNNNNNNNCNNTNNNNNN

>TSA1_ForwardRead
NNNNNNNNNNNNTNNGNAANNCGTACANGTANCCGTANCTCTTANCNNGGTAGCCNGTANCT
CNNNNNAGGTANCCNNANGTCGGGGTGAGTAACACGTGNGTAACCTGCCGATCCCAAGGGG
ATAACACTTGGAAACAGGTGCTAATACCGTATAACAATCGAAACCGCATGGTTTTGATTTGA
AAGGCGCTTTCGGGTGTCGCTGATGGATGGACCCGCGGTGCATTAGCTAGTTGGTGAGGTAA
CGGCTCACCAAGGCCACGATGCATAGCCGACCTGAGAGGGTGATCGGCCACATTGGGACTG
AGACACGGCCCAAACTCCTACGGGAGGCAGCAGTAGGGAATCTTCGGCAATGGACGAAAGT
CTGACCGAGCAACGCCGCGTGAGTGAAGAAGGTTTTCGGATCGTAAAACTCTGTTGTTAGAG
AAGAACAAGGATGAGAGTAACTGTTCATCCCTTGACGGTATCTAACCAGAAAGCCACGGCT
AACTACGTGCCAGCAGCCGCGGTAATACGTAGGTGGCAAGCGTTGTCCGGATTTATTGGGCG
TAAAGCGAGCGCAGGCGGTTTCTTAAGTCTGATGTGAAAGCCCCCGGCTCAACCGGGGAGG
GTCATTGGAAACTGGGAGACTTGAGTGCAGAAGAGGAGAGTGGAATTCCATGTGTAGCGGT
GAAATGCGTAGATATATGGAGGAACACCAGTGGCGAANGCGGCTCTCTGGTCTGTAACTGA
CGCTGANGCTCGAAAGCGTGGGGAGCAAACAGGATTAGATACCCTGGTAGTCCACGCCGTA

AACGATGAGTGCTAAGTGTTGGAGGGTTTCCGCCCTTCAGTGCTGCAGCTAACGCATTAAGC
ACTCCGCCTGGGGAGTACGACCGCAAGGTTGAAACTCAAAGGAATTGACGGGGGCCCGCAC
AAGCGGTNGNNCATGTNNTTAATTCGAAGCAACGCGANAANNTTACCNNNCTTGACNTCCN
TTGACCNCTCTAGANNNNAGANCTTCCCTNNGGGGNAAANTGNCNGNNNNCANNNNNTCGN
CAGCNNNNNGTNNNNNATNTNNNNNNNTCCGNANNANNNNNNNNNNNATNTANTNCNTCNTCA
NNNN

>TSA1_ReverseRead
NNNNNNNNNNNNNNNNNNNNNNNGCTACNTNGTTACGACTNANGGCTACCTTGTTACNACNN
AGTGCTACCTTGNNACGATGTANCGGGCGGTGGGTACAAGGNACGGGAACGTATTCACCGC
GGAGTGCTGATCCGCGATTACTAACGATTCCGGCTTCATGCAGGCGAGTTGCAGCCTGCAAT
CCGAACTGAGAGAAGCTTTAAGAGATTAGCTTAGCCTCGCGACTTCGCAACTCGTTGTACTT
CCCATTGTAGCACGTGTGTAGCCCAGGTCATAAGGGGCATGATGATTTGACGTCATCCCCAC
CTTCCTCCGGTTTGTCACCGGCAGTCTTGCTAGAGTGCCCAACTGAATGATGGCAACTAACA
ATAAGGGTTGCGCTCGTTGCGGGACTTAACCCAACATCTCACGACACGAGCTGACGACAACC
ATGCACCACCTGTCACTTTGCCCCCGAAGGGGAAGCTCTATCTCTAGAGTGGTCAAAGGATG
TCAAGACCTGGTAAGGTTCTTCGCGTTGCTTCGAATTAAACCACATGCTCCACCGCTTGTGCG
GGCCCCCGTCAATTCCTTTGAGTTTCAACCTTGCGGTCGTACTCCCCAGGCGGAGTGCTTAAT
GCGTTAGCTGCAGCACTGAAGGGCGGAAACCCTCCAACACTTAGCACTCATCGTTTACGGCG
TGNACTACCAGGGTATCTAATCCTGTTTGCTCCCCACGCTTTCGAGCCTCAGCGTCAGTTACA
GACCAGAGAGCCGCCTTCGCCACTGGTGTTCCTCCATATATCTACGCATTTCACCGCTACAC
ATGGNAATTCCACTCTCCTCTTCTGCACTCAAGTCTCCCAGTTTCCAATGACCCTCCCCGGGT
TGAGCCGGGGGCTTTCACATCAGACTTAANAAACCGCCTGCGCTCGCTTTACGCCNNNAAAT
CNNNANNNGCTTGCNNCCTACGTATTACNNNGCTGCTGGCACGTAGTTAGCCNNNGNTTNCT
NNNNNNNNNCNTCAGGGANGAANANTNCTCTCANNCNNNNNTNNNNTAACANCNNNANTTTT
ACGANCNAAAACTTNNNNNNNNNNNNNNNGNNNNNNNNNNNGNNCNANANTTTNNNNCNNN

>TSA2_ForwardRead
NNNNNNNNNNNNNNNNNANNCNTGNCAGTCNAACGGTAGCACAGAGGAGCGTTGCTCCTA
TGGGTGACGAGTGGCGGACGGGTGAGTAATGTCTGGGAAACTGCCCGATGGAGGGGGATAA
CTACTGGAAACGGTAGCTAATACCGCATAACGTCGCAAGACCAAAGAGGGGGACCTTCGGG
CCTCTTGCCATCGGATGTGCCCAGATGGGATTAGCTAGTAGGTGGGGTAACGGCTCACCTAG
GCGACGATCCCTAGCTGGTCTGAGAGGATGACCAGCCACACTGGAACTGAGACACGGTCCA
GACTCCTACGGGAGGCAGCAGTGGGGAATATTGCACAATGGGCGCAAGCCTGATGCAGCCA
TGCCGCGTGTATGAAGAAGGCCTTCGGGTTGTAAAGTACTTTCAGCGAGGAGGAAGGTGTTG
TGGTTAATAACCGCAGCAATTGACGTTACTCGCAGAAGAAGCACCGGCTAACTCCGTGCCAG
CAGCCGCGGTAATACGGAGGGTGCAAGCGTTAATCGGAATTACTGGGCGTAAAGCGCACGC
AGGCGGTCTGTCAAGTCGGATGTGAAATCCCCGGGCTCAACCTGGGAACTGCATCCGAAACT
GGCAGGCTAGAGTCTTGTAGAGGGGGGTAGAATTCCAGGTGTAGCGGTGAAATGCGTAGAG
ATCTGGAGGAATACCGGTGGCGAAGGCGGCCCCCTGGACAAAGACTGACGCTCNGTGCGAA
AGCGTGGGGAGCAAACAGGATTAGATACCCTGGTAGTCCACGCCGTAAACGATGTCGACTN
NGAGGTTGTGCCCTTGAGGCGTGGNTTCCGGAGCTAACGCGTTAAGTCGACCGCCTGGGGA
GTACGGCCGCAAGGTTAAAACTCAAATGAATTNGANGGGGCCCGCACAAGCGGNGGAGCAT
GTGGNTTAATTCGATGCAACGCGAAGAANCTTACCTACTCTTGACATCCANNAGAAGTTTGC
NNANATGCNAACGTGCCTTNCGGGANTCNGANNACNNNGCTGCATGNNGTCGTCAGCTCGN
GNNGNNNAANNNTNNNNNNTNCNNNANGANNGCNNCCNNNCCNTNNNTGCNNNNNNTTCN
GNCGGNNNNCAAAGNNN

>TSA2_ReverseRead

NNNNNNNNNNNNTNNNNNNNNNANGNNNNNCGNNNTCCCGNNNNNNNNANCTACCTACTTCTT
TTAGCAACCCACTCCCATGGTGTGACGGGCGGTGTGTACAAGGCCCGGGAACGTATTCACCG
TGGCATTCTGATCCACGATTACTAGCGATTCCGACTTCATGGAGTCGAGTTGCAGACTCCAA
TCCGGACTACGACATACTTTATGAGGTCCGCTTGCTCTCGCGAGGTCGCTTCTCTTTGTATAT
GCCATTGTAGCACGTGTGTAGCCCTACTCGTAAGGGCCATGATGACTTGACGTCATCCCCAC
CTTCCTCCAGTTTATCACTGGCAGTCTCCTTTGAGTTCCCGACCGAATCGCTGGCAACAAAGG
ATAAGGGTTGCGCTCGTTGCGGGACTTAACCCAACATTTCACAACACGAGCTGACGACAGCC
ATGCAGCACCTGTCTCAGAGTTCCCGAAGGCACGTTCGCATCTCTGCAAACTTCTCTGGATG
TCAAGAGTAGGTAAGGTTCTTCGCGTTGCATCGAATTAAACCACATGCTCCACCGCTTGTGC
GGGCCCCCGTCAATTCATTTGAGTTTTAACCTTGCGGCCGTACTCCCCAGGCGGTCGACTTAA
CGCGTTAGCTCCGGAAGCCACGCCTCAAGGGCACAACCTCCAAGTCGACATCGTTTACGGCG
TGGACTACCAGGGTATCTAATCCTGTTTGCTCCCCACGCTTTCGCACCTGAGCGTCAGTCTTT
GTCCAGGGGGCCGCCTTCGCCACCGGTATTCCTCCAGATCTCTACGCATTTCACCGCTACACC
TGGAATTCTACCCCCCTCTACAAGACTCTAGCCTGCCAGTTTCGGATGCAGTTCCCAGGTTGA
GCCCGGGGATTTCACATCCGACTTGACAGACCGCCNGCGTGCGCTTTACGCCCAGTAATTCC
GATTAACGCTTGNNCCCTCCGTATTACCGCGGCTGCTGGNACGGANTTAGCCNGNGNTTNTT
CTGCNAGTAACGTCANTNNNNGNNGTTANTNNNANNNNNNTTCNNNCNCTGAAGTACTTTN
NNACCNNNNNNNTNNNNNNNNNNNNNNNATGNTGCATCNGCTTNNNNNNCCATNNNNGCANNNN
NNNNNN

>TSA3_ForwardRead
NNNNNNNNNNNNNNNNNNCNCNTGACAGTNNAGCGGTAGCACAAGAGAGCNTTGCTCTCANG
GGTGACGAGCGGCGGACGGGTGAGTAATGTCTGGGAAACTGCCTGATGGAGGGGGATAACT
ACTGGAAACGGTAGCTAATACCGCATGACGTCTTCGGACCAAAGTGGGGGACCTTCGGGCC
TCACGCCATCAGATGTGCCCAGATGGGATTAGCTAGTAGGTGGGGTAATGGCTCACCTAGGC
GACGATCTCTAGCTGGTCTGAGAGGATGACCAGCCACACTGGAACTGAGACACGGTCCAGA
CTCCTACGGGAGGCAGCAGTGGGGAATATTGCACAATGGGCGCAAGCCTGATGCAGCCATG
CCGCGTGTATGAAGAAGGCCTTCGGGTTGTAAAGTACTTTCAGCGAGGAGGAAGGCATTAA
GGTTAATAACCTTGGTGATTGACGTTACTCGCAGAAGAAGCACCGGCTAACTCCGTGCCAGC
AGCCGCGGTAATACGGAGGGTGCAAGCGTTAATCGGAATTACTGGGCGTAAAGCGCACGCA
GGCGGTTTGTTAAGTCAGATGTGAAATCCCCGAGCTTAACTTGGGAACTGCATTTGAAACTG
GCAAGCTAGAGTCTTGTAGAGGGGGGTAGAATTCCAGGTGTAGCGGTGAAATGCGTAGAGA
TCTGGAGGAATACCGGTGGCGAAGGCGGCCCCCTGGACAAAGACTGACGCTCANGTGCGAA
AGCGTGGGGAGCAAACAGGATTAGATACCCTGGTAGTCCACGCTGTAAACGATGTCGACTT
GGAGGTTGTGCCCTTGAGGCGTGGNTTCCGGAGCTAACGCGTTAAGTCGACCGCCTGGGGA
GTACGGCCGCAAGGTTAAAACTCAAATGAATTGANGGGGGCCCGCACAAGCGGTGGANCAT
GNGGTTTAATTCGATGCAACGCGAANAANCNTTACCTACTCTTGACNTCCAGAGAATTTGCT
AGAGATAGCTTAGTGCCTTNCGGGNNTCTGANANNGNNGCTGCATGGNTGTCGTCAGCTCN
NNNNNNGAAATGTTNGGNNNNGTCNNNANNANNCGNNNNNNNATNNNNNNNNTNCNGCNNNT
NNNGGNNGGGNNNCAAGGANACTNNNNNNNNNNNNNNNANNANGNNGGGNNNNNNNNNCNNN
NNNNNNNNNNNNNNNNTNNNNNNN

>TSA3_ReverseRead
NNNNNNNNNNNNNNNNNNNNNNNNTNNNCNNNNTCCGANTANTTAGAGCATNACCTNACTNTCTA
TTTAGCAACCCACTCCCATGGTGTGACGGGCGGTGTGTACAAGGCCCGGGAACGTATTCACC
GTAGCATTCTGATCTACGATTACTAGCGATTCCGACTTCATGGAGTCGAGTTGCAGACTCCA
ATCCGGACTACGACATACTTTATGAGGTCCGCTTGCTCTCGCGAGTTCGCTTCTCTTTGTATA
TGCCATTGTAGCACGTGTGTAGCCCTACTCGTAAGGGCCATGATGACTTGACGTCATCCCCA
CCTTCCTCCGGTTTATCACCGGCAGTCTCCTTTGAGTTCCCANCATTACNNGCTGGCAACAAA

GGATAAGGGTTGCGCTCGTTGCGGGACTTAACCCAACATTTCACAACACGAGCTGACGACA
GCCATGCAGCACCTGTCTCAGAGTTCCCGAAGGCACTAAGCTATCTCTAGCAAATTCTCTGG
ATGTCAAGAGTAGGTAAGGTTCTTCGCGTTGCATCGAATTAAACCACATGCTCCACCGCTTG
TGCGGGCCCCCGTCAATTCATTTGAGTTTTAACCTTGCGGCCGTACTCCCCAGGCGGTCGACT
TAACGCGTTAGCTCCGGAAGCCACGCCTCAAGGGCACAACCTCCAAGTCGACATCGTTTACA
GCGTGNACTACCAGGGTATCTAATCCTGTTTGCTCCCCACGCTTTCGCACCTGANCGTCAGTC
TTTGTCCAGGGGGCCGCCTTCGCCACCGGTATTCCTCCAGATCTCTACGCATTTCACCGCTAC
ACCTGGAATTCTACCCCCCTCTACAAGACTCTAGCTTGNCAGTTTCAAATGCAGTTCCCAAGT
TAAGCTCGGGGATTTCACATCTGACTTAANNAACCGCCNGCGTGCGCTTTACGCCNAGTAAT
TCCCGATTAACGCTTGCACCCTNCGTATTACCGCGGCTNCTGGNNCGGANTTAGCNNGTGNT
NNNTCNNNNAGTAACGTCANCACCNANNNANTNNCNNNNNNGNNNNNNNNCTGAAAGTAC
TTANANNNNANNTNTNNNNNNNNNCGNATGNNNNATCNGNTNNNNCCCANNNNNCANNTNC
CCNNNGCNNCNNCCGNNNNNNNNNNNN
