# Supplementary material for: Human norovirus binding to select bacteria representative of the human gut microbiota
Source: PLoS One. 2017 Mar 3;12(3):e0173124. doi: 10.1371/journal.pone.0173124 (PMC5336261; doi:10.1371/journal.pone.0173124)
Supplement: S1 Table — Different samples of human stool were streak plated and grown in both aerobic and anaerobic conditions. Specific colonies were isolated, further cultured and their 16S rRNA region sequenced. Below is a summary table of the top sequence isolates selected for further analysis. (PDF) [file pone.0173124.s002.pdf]

Supplemental Information

S1 Table. Top matches for 16S rRNA sequences of bacteria isolated from human stool samples. Different samples of human stool were streak plated and grown in both aerobic and anaerobic conditions. Specific colonies were isolated, further cultured and their 16S rRNA region sequenced. Below is a summary table of the top sequence isolates selected for further analysis.

Isolate Name	Top Matches	Accession Number	Length (bp)	Query Coverage (%)	Identity (%)
BA2	*Enterococcus casseliflavus* EC20, complete genome	NC_020995.1	868	68	97
	Enterococcus faecium Aus0085, complete genome	NC_021994.1	858	67	97
	Enterococcus faecium NRRL B-2354, complete genome	NC_020207.1	858	67	97
BAB1	*Bacillus toyonensis* BCT-7112, complete genome	NC_022781.1	918	78	97
	Bacillus thuringiensis YBT-1518, complete genome	NC_022873.1	919	78	97
	Bacillus thuringiensis serovar thuringiensis str 1S5056	NC_020376.1	919	78	97
BAB2	*Klebsiella oxytoca* E718 complete genome	NC_018106.1	1010	83	96
TSA1	*Enterococcus faecium* Aus0085, complete genome	NC_021994.1	915	82	97
	Enterococcus faecium NRRL B-2354, complete genome	NC_020207.1	915	82	97
	Enterococcus faecilis D32, complete genome	NC_018221.1	916	82	95
TSA2	*Citrobacter freundii* CFNIH1, complete genome	CP007557.1	944	84	98
TSA3	*Hafnia alvei* FB1 complete genome	CP009706.1	1011	85	97
